# Utilisation of cervical cancer screening among women living with HIV at Kenya’s national referral hospital

**DOI:** 10.4102/sajhivmed.v23i1.1353

**Published:** 2022-04-25

**Authors:** James M. Kangethe, Aliza Monroe-Wise, Peter N. Muiruri, James G. Komu, Kenneth K. Mutai, Mirriam M. Nzivo, Jillian Pintye

**Affiliations:** 1 Comprehensive Care Center, HIV Medicine, Kenyatta National Hospital, Nairobi, Kenya; 2 Department of Medical Microbiology and Immunology, Faculty of Health Sciences, University of Nairobi, Nairobi, Kenya; 3 Department of Global Health, International AIDS Research and Training Program, University of Washington, Seattle, United States of America; 4 Department of Global Health, University of Washington, Seattle, United States of America; 5 Department of Medical Laboratory Sciences, College of Health Sciences, Jomo Kenyatta University of Agriculture and Technology, Nairobi, Kenya; 6 Department of Biological Sciences, Jomo Kenyatta University of Agriculture and Technology, Nairobi, Kenya; 7 Department of Biological Sciences, University of Embu, Embu, Kenya; 8 Department of Biobehavioral Nursing and Health Informatics, University of Washington, Seattle, United States of America

**Keywords:** women living with HIV, cervical cancer, Kenya, utilisation, integrated cervical cancer screening

## Abstract

**Background:**

In 2009, Kenyatta National Hospital (KNH) integrated cervical cancer screening within HIV care using visual inspection with acetic acid (VIA) and Pap smear cytology.

**Objectives:**

We evaluated utilisation of cervical cancer screening and human papillomavirus (HPV) vaccination among women living with HIV (WLHIV) receiving HIV care at KNH.

**Method:**

From November 2019 to February 2020, WLHIV aged ≥ 14 years were invited to participate in a survey following receipt of routine HIV services. We assessed awareness of cervical cancer, uptake of cervical cancer screening, uptake of the HPV vaccine, and barriers to utilisation of these services. In a subset of survey participants, focus group discussions (FGDs) were also conducted to identify screening barriers.

**Results:**

Overall, 305 WLHIV participated in the survey. Median age was 36 years (interquartile range [IQR]: 28–43), 41% were married, and 38% completed secondary education. Most (90%) had HIV RNA < 1000 copies/mL. Awareness of cervical cancer was high (84%), although only 45% of WLHIV had screened for cervical cancer at the referral hospital and only 13% knew how to prevent high-risk HPV. No participants had received an HPV vaccination. Older age, higher education, and knowledge of the HPV vaccine were associated with higher likelihood of cervical cancer screening (*P* < 0.05). In FGDs, barriers to utilising the services included user fees, fear of the procedure impacting fertility, age and gender of the provider, and long waiting times.

**Conclusion:**

Despite integration with HIV services, the utilisation of cervical cancer screening was low among WLHIV and implementation barriers contributed to low utilisation.

## Introduction

Cervical cancer is the second most common cancer among women in sub-Saharan Africa (SSA).^1^ In Kenya, over 5000 new cervical cancer cases occur annually resulting in over 3000 deaths.^2^ Over half of Kenyan women living with HIV (WLHIV) are infected with a high-risk human papillomavirus (HR-HPV) genotype^3^ which contributes to higher cervical cancer incidence and mortality among Kenyan WLHIV.^4^ The Kenyan HIV prevalence among women of reproductive age is 6.2%.^5^ The World Health Organization (WHO) recommends integrating cervical cancer prevention services at primary, secondary, and tertiary levels within HIV care programmes.^6^ This includes the provision of HPV vaccination to eligible adolescent girls and young women (AGYW) combined with health education on risk factors for cervical cancer,^7,8^ and regular screening for early detection among at risk women,^9,10^ and treatment with surgery, cryotherapy, radiotherapy, or chemotherapy, when necessary. In SSA, screening is commonly practised using non-cytological screening methods such as visual inspection with acetic acid (VIA) or Lugol’s iodine (VILI). Visual inspection with acetic acid is more frequently used in SSA since acetic acid is readily available locally.^11^ Despite the high burden of cervical cancer among WLHIV in SSA, utilisation of cervical cancer prevention services remains lower than 5%.^12^ The Kenyan cervical cancer screening guidelines recommend screening for women aged 25 years and above or if they are already sexually active. Screening interval is five years for women who test negative for HIV. However, for WLHIV whose screening results are negative, repeat screening should be done annually (if initial screening was done with VIA or Pap smear) and after two years if HPV molecular testing was used and tested negative.

Screening by VIA for WLHIV has been recommended since 2009 (WHO Guidelines, 2006), with Pap smear indicated as the confirmatory test for those with positive VIA. According to cervical cancer guidelines published in 2018, all WLHIV should be screened annually upon testing negative with VIA or Pap smear. However, if HPV screening was used and tested negative, then rescreening should be done after 2 years.^13^ In Kenya, the cervical cancer screening uptake is higher among WLHIV (14%) compared to 3% among HIV-negative women.^14^ The Kenyan HPV vaccination programme launched in 2019 targeting HIV-negative girls aged 10–15 years and adolescent girls and young adult WLHIV aged 10–24 years. The type of HPV vaccine available to protect against HPV infection is Gardasil which is a quadrivalent vaccine and protective against HPV types 6, 11, 16 and 18.^13^ More data are needed to understand utilisation within the context of integrated service delivery in HIV care programmes to guide implementation improvement efforts.

According to Kenyan national guidelines, annual cervical cancer screening for WLHIV 25–49 years is recommended; however, age is not limiting and any sexually active WLHIV is eligible for screening.^13^ In 2009, Kenyatta National Hospital (KNH) integrated cervical cancer preventive services within its Comprehensive Care Center (CCC) for HIV care and treatment. To date, no evaluation of cervical cancer service utilisation has been conducted at the referral hospital. We aimed to evaluate the utilisation of cervical cancer prevention services and identify barriers to utilisation among WLHIV enrolled for care through a cross-sectional survey complemented by focus group discussions (FGDs) among a subset of participants. The overall aim of our evaluation is to guide implementation efforts to improve delivery and utilisation of cervical cancer prevention services by WLHIV in SSA.

## Research methods and design

### Study design

This was a mixed methods study that included both exploratory qualitative analysis and a cross-sectional quantitative survey among consecutive sample of WLHIV.

### Study site, population and recruitment

Based on programmatic data, the clinic annually serves approximately 10 000 patients on HIV treatment, with roughly half being WLHIV of reproductive health age. All WLHIV seeking services at the CCC who were ≥ 14 and < 25 years, aware of their HIV status, and willing and able to consent were eligible to enrol in our survey.

For women aged ≥ 25 years, there was an additional eligibility criterion that they received care at the CCC for ≥ 1 year. According to the Kenyan cervical cancer screening guidelines (Ministry of Health Kenya 2018),^13^ WLHIV aged 25 years and above should be screened annually. To avoid enrolling WLHIV newly enrolled into HIV care, we restricted sampling to women > 25 years who were in care for > 1 year to ensure they had adequate time to meet the annual screening guidelines. The sample size was calculated using Fisher’s formula for cross-sectional studies at 95.0% confidence interval, with a margin error of 2.5% and a 5.0% utilisation of cervical cancer prevention services from a study conducted in low- and middle-income countries.^15^

In the CCC, the cervical cancer prevention services that were integrated with the HIV care include cervical cancer screening using VIA and Pap smear cytology (if abnormalities are detected with VIA), and referral to treatment at the gynaecological specialised clinic, when indicated. While VIA is offered at no cost to eligible women, if Pap smear cytology is indicated, there is a cost implication to be incurred by the WLHIV. The HPV vaccination for AGYW < 25 years is also recommended and is only offered in a different unit from the CCC. The HPV vaccination is available at a cost of more than $50.00 (United States dollars) for eligible AGYW. Both VIA and Pap cytology are done by nurses based at CCC who received specialised training and mentorship on cervical cancer screening.

Patients are referred to the national referral hospital due to a variety of health complications including HIV. Women living with HIV residing in the metro Nairobi area may also seek routine HIV services at KNH without referral from another facility. Based on the Kenya Ministry of Health guidelines for HIV care and treatment, all persons diagnosed with HIV are started on first-line antiretroviral therapy (ART) immediately. Currently, Dolutegravir (DTG) based regimens are recommended for the first-line and second-line ART.^16,17^ Tuberculosis (TB) screening and management for persons living with HIV are integrated in the HIV clinic. All WLHIV are screened symptomatically for sexually transmitted infections (STIs) and treated per Kenyan syndromic management guidelines. According to the Kenyan guidelines (Ministry of Health Kenya 2018), VIA is recommended annually for WLHIV aged 25–49 years and for those who are less than 25 years of age but are sexually active. Women living with HIV are scheduled to attend their routine visits every 3–6 months. Once the services are offered, the patient’s details are entered into electronic medical records at the clinic and the patient issued with a cervical cancer screening card indicating the current results, intervention recommended, if any, and the next scheduled screening. From November 2019 to February 2020, consecutive sampling was used to screen WLHIV to participate in the survey following receipt of HIV services. One research assistant (RA) who was based at the CCC every weekday morning between 09:00 and 12:00 invited WLHIV to join the study following routine visits. Women living with HIV were approached at the triage area of the CCC and the study was explained to them by the RA. Those who were willing to participate were consented and counselled on the benefits of cervical cancer screening. On average, the RA approached 7 WLHIV each day. A subset of survey participants was invited to join FGDs to understand barriers to accessing cervical cancer preventive services. Purposive sampling was used to invite participants based on age categories to create age-specific FGDs, each with six participants: 14–19 years (1 FGD), 20–24 years (1 FGD), and 25–49 years (2 FGDs).

## Data collection and analysis

### Quantitative survey

Structured, pre-tested paper-based questionnaires captured socio-demographic characteristics and factors influencing the utilisation of cervical cancer services. Knowledge, attitudes, and current practices regarding cervical cancer screening were also assessed (Appendix 1). The questionnaire was piloted among WLHIV seeking care at the study site before study recruitment started. During the pilot period, the appropriateness of the tool was assessed by how well respondents understood the questions and if the responses were congruent with the information we planned to obtain. The questionnaire was administered in English and clarifications were made by the RA when requested by respondents. Utilisation was defined as self-reported prior receipt of HPV vaccination (for participants < 25 years) or cervical cancer screening at CCC using VIA or Pap smear cytology. The satisfaction of participants with healthcare workers’ (HCW) counselling services was defined using a numerical rating scale: 1 = poor, 2 = average, 3 = good, 4 = excellent. The satisfaction of the HCW’s counselling was based on the current HIV clinic visit at KNH CCC. Clinical data were abstracted from medical records upon obtaining consent. The relationships between participant characteristics and utilisation of cervical cancer services were evaluated using multivariate logistic regression models. We determined a priori to adjust all multivariate models for age (years) and education level (primary and below vs above primary education). Data were analysed using Statistical Package for Social Sciences (SPSS) version 23.0.

### Qualitative focus group discussions

Four (4) FGDs, each with six participants and lasting 30–40 min, were conducted, recorded, and transcribed. English and Kiswahili languages were used interchangeably and later translated to English. Qualitative data were deductively analysed and categories were constructed for content themes. Patterns of the content appearing repeatedly in the data formed the basis for themes. Themes were grouped to provide an integrated explanation of why participants utilised or did not utilise cervical preventive services at the CCC.

### Ethical considerations

An application for full ethical approval was made to the University of Nairobi, Kenyatta National Hospital Ethics and Review Committee (UON/KNH ERC) and ethics approval was received in October 2019. The ethics approval number is P109/02/2019. All procedures performed in studies involving human participants were in accordance with the ethical standards of the institutional and national research committees and with the 1964 Helsinki Declaration and its later amendments or comparable ethical standards. Participants < 18 years signed an assent form after obtaining parental or guardian written consent. Participants aged 18 years and above provided written informed consent. Consent forms were available in participants’ preferred language, either English or Kiswahili.

## Results

**FIGURE 1:**
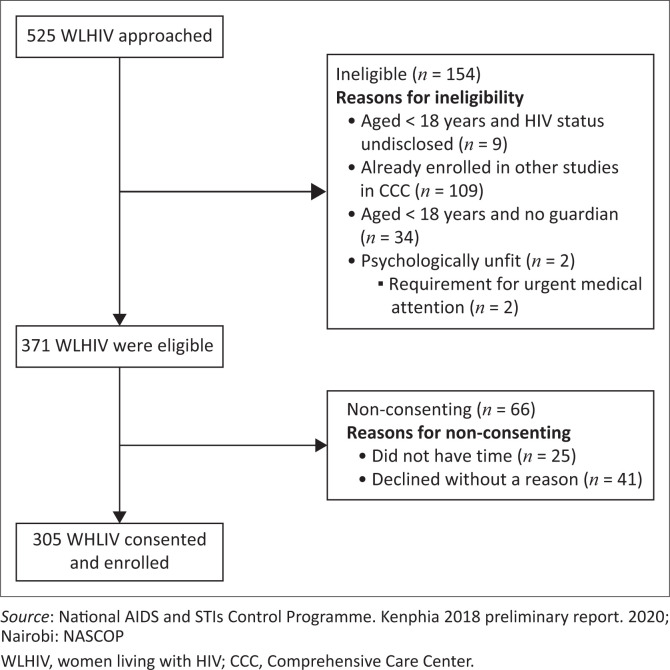
Flow diagram of the enrolment process.

In total, 525 WLHIV were approached and screened for eligibility. Of the 371 eligible WLHIV, 305 consented and were enrolled into the study (Figure 1).

Of the 371 WLHIV who were eligible to participate, 66 WLHIV did not consent to participate in our study as they had no time or declined without a reason.

The median age was 36 years (interquartile range [IQR]: 28–43), 10.5% of participants were < 18 years, 41% of participants were married, and 38% had a secondary level of education (as shown in Table 1). The median CD4 count was 547 cells/mm^3^ (IQR: 351–698). Most (90.7%) were virally suppressed with < 1000 HIV RNA copies/mL.

**TABLE 1:** Participant characteristics, awareness, and utilisation of cervical cancer services.

Variable	Frequency (*n*)	Percentage	Median	IQR
**Age (years)**	-	-	36	28–43
**Marital status**				
Single	99.0	32.5	-	-
Married	125.0	41.0	-	
Divorced	47.0	15.4	-	
Widowed	34.0	11.1	-	
**Education level**			
None	18.0	5.9	-	-
Primary	72.0	23.6	-	-
Secondary	117.0	38.4	-	-
College	98.0	32.1	-	-
**Employment status**				
Unemployed	100.0	32.8	-	-
Employed	205.0	67.2	-	-
Smokes cigarettes	13.0	4.3	-	-
STI infection treated in the last 6 months†	31.0	10.2	-	-
CD4 count at the last HIV care (*n* = 227) (cells/mL)	-	-	547	351–698
**Viral load at the last HIV care (*n* = 257)**				
< 1000 copies/mL	233	90.7	-	-
> 1000 copies/mL	24	9.3	-	-
Knowledge of cervical cancer	257	84.3	-	-
Screened for cervical cancer in KNH CCC	136	44.6	-	-
Received cervical cancer screening results (*n* = 136)	131	96.3	-	-
**Turnaround time for cervical cancer screening results (*n* = 131)**				
1–2 weeks	112	85.5	-	-
1 month	10	7.6	-	-
2 months	5	3.8	-	-
3–6 months	4	3.1	-	-
Knowledge of how to prevent high-risk HPV infections (*n* = 305)	40	13.1	-	-
Ever heard of HPV vaccine	152	49.8	-	-
Received HPV vaccination	0	0.0	-	-
Received health education from HCW on cervical cancer preventive services	150	49.2	-	-
**HCW education rating (*n* = 150)**				
Poor	18	12.0	-	-
Average	58	38.7	-	-
Good	60	40.0	-	-
Excellent	14	9.3	-	-
Smokes cigarettes	13	4.3	-	-

*Source*: National AIDS and STIs Control Programme. Kenphia 2018 preliminary report. 2020; Nairobi: NASCOP.

*N* = 305.

CCC, Comprehensive Care Center; IQR, interquartile range; HCW, healthcare workers; HPV, human papillomavirus; WLHIV, women living with HIV; STI, sexually transmitted infection.

† , STIs in the last 6 months were assessed via self-report and abstraction from medical records.

Few participants (13.1%) were able to explain how HR-HPV infections are transmitted and ways to prevent HR-HPV transmission. Half (49.2%; *n* = 49.2) of the women received health education on cervical cancer at their current clinic visit. Less than half of the participants (40.0%) rated cervical cancer-related health education offered by the HCW to be ‘good’ or ‘excellent’ compared to 60.0% who reported ratings of ‘average’ or ‘poor’.

Only 44.6% of participants previously received cervical cancer screening at the CCC. No participants had received the HPV vaccination. Among the WLHIV who had been screened for cervical cancer at the CCC (*n* = 136), 16.1% were referred to a different clinic away from the CCC for further intervention or vaccination, if eligible. Among WLHIV who had abnormalities detected via VIA, 85.5% went for Pap smear cytology and received results within two weeks.

As shown in Table 2, WLHIV screened for cervical cancer were more frequently ≥ 36 years compared to those not screened (68% vs 39%, adjusted odds ratio [AOR] = 3.2, 95% confidence interval [CI]: 2.0–5.1, *P* < 0.001). Compared to WLHIV not screened for cervical cancer, those screened were more likely to have at least a primary level of education or above (81% vs 62%, AOR = 2.4, 95% CI: 1.4–4.3, *P* = 0.002). Women living with HIV screened for cervical cancer also were more likely to have heard of the HPV vaccine compared to unscreened WLHIV (63% vs 38%, % AOR = 2.0, 95% CI: 1.2–3.5, *P* = 0.014). There were no other differences detected between WLHIV who were screened for cervical cancer and those who were not.

**TABLE 2:** Correlates of the utilisation of cervical cancer screening services among women living with HIV.†^5^

Variable	Screened (*n* = 136)		Unscreened (*n* = 169)		OR	95% CI	*P*	AOR‡	95% CI	*P*
	*n*	%	*n*	%						
**Age**						2.0–5.3			2.0–5.1	< 0.001
< 36	43	31.9	102	60.7	1.0;3.3	-	-	1.0;3.2	-	
36+	93	68.1	67	39.3	-	-	< 0.001	-	-	
**Education**								-	1.4–4.3	0.002
Primary and below	26	19.1	64	37.9	1.0;2.6	1.5–4.4	-	1.0;2.4	-	
Above primary	110	80.9	105	62.1	-	-	< 0.001	-	-	
**Marital status**						0.5–1.2		-	-	
Married	61	44.9	64	37.9	1.0;0.7	-	-	-	-	
Unmarried	75	55.1	105	61.1	-	-	0.218	-	-	
**Viral load at last HIV care visit *n* = 257**						0.8–4.5		-	-	
< 1000	112	93.3	121	88.3	1.9;1.0	-	0.168	-	-	
> 1000	8	6.7	16	11.7	-	-	-	-	-	
**Ever heard of HPV vaccine**						1.6–4.0			1.2–3.5	0.014
Yes	85	62.5	67	39.6	-	-	-	-	-	
No	51	37.5	102	60.4	2.5;1.0	-	0.001	2.0;1.0	-	
**Been treated for an STI in the last 6 months**						0.2–0.9			0.3–2.2	0.747
Yes	8	5.9	23	13.6	0.4;1.0	-	0.026	0.9;1.0	-	
No	128	94.1	146	86.4	-	-	-	-	-	
**Employment status**						2.4–7.0			1.0–3.3	0.059
Unemployed	23	16.9	77	45.6	1.0;4.1	-	-	1.0;1.8	-	
Employed	113	83.1	92	54.4	-	-	< 0.001	-	-	
**Have living children *n* = 267**						2.0–7.8			0.9–4.6	0.082
Yes	112	89.6	97	68.3	4.0;1.0	-	< 0.001	2.1;1.0	-	
No	13	10.4	45	31.7	-	-	-	-	-	

*Source*: National AIDS and STIs Control Programme. Kenphia 2018 preliminary report. 2020; Nairobi: NASCOP.^5^

HPV, human papillomavirus; STI, sexually transmitted infection; OR, odds ratio; AOR, adjusted odds ratio; 95 % CI, 95% confidence interval.

† , Cervical cancer screening was done using either using VIA or Pap smear cytology.

‡ , All multivariate models were adjusted for age (years) and level of education (primary and below or above primary).

### Qualitative perspectives on cervical cancer screening within HIV care

In FGDs, several participants expressed their concerns that cervical cancer screening was invasive and painful. This prompted them to decline screening. Even after an explanation by a HCW, women expressed concerns about how painful the procedure is. One participant said:

‘I have never gone for the screening, although my doctor recommended it several times. I fear it will be very painful’. (Participant 5, female, 34 years old)

Other participants, especially adolescents, were fearful that administration of the HPV vaccine or cervical cancer screening would interfere with their future fertility. A participant said:

‘The thing that really gets me concerned about this HPV vaccine and cervical cancer screening is the uncertainty of how they relate with my future fertility’. (Participant 24, female, 23 years old)

Several participants were not comfortable with male providers, indicating that their culture did not allow them to be seen by a male provider for such invasive procedures. One participant reported:

‘It matters whether it’s a male or a female [*provider*] because if a female tells you she will do the screening, you will be at ease and consent to screening as opposed to if the screening was done by a male as you feel victimised. Old mature ladies take their time to address most of your concerns before they start attending to you, unlike the young service providers’. (Participant 9, female, 41 years old)

In some instances, (e.g. stock-outs, etc.), screening is not conducted in CCC and WLHIV are referred to the another unit in the referral hospital. This additional navigation through the hospital increases loss to follow-up as expressed by one participant:

‘I was sent by the doctor to be screened at a different clinic from CCC. I went and waited for two and a half hours and still, no one came to attend to me. Later, I was rebooked to come for screening 10 days later. My work schedule isn’t flexible’. (Participant 3, female, 38 years old)

Some women did not perceive themselves to be at risk due to the absence of cervical pain or abnormalities. Adolescents believed that cervical cancer was a disease of older women. Others claimed they had only one sexual partner and thus were not at risk for cervical cancer. Many women believed that cervical cancer is a curse from God with no cure or a punishment from the ancestors due to the wrongdoings as expressed by one participant:

‘Cervical cancer is a curse from God, even if you know you have it, nothing will change and no treatment will fight it. You will end up being completely depressed’. (Participant 2, female, 49 years old)

### Cost of the cervical cancer prevention services

Unlike HIV services which are free, payment is required for Pap smear cytology, if indicated following VIA. Moreover, HPV vaccination is offered at cost. Thus, user fees limit utilisation of prevention services as described by one participant:

‘Cervical cancer vaccination is not offered in CCC and in the vaccination centre it’s not free, unlike ARVs [*antiretrovirals*] and viral load testing. I can’t afford to pay for it’. (Participant 22, female, 19 years old)

## Discussion

Our study of utilisation of cervical cancer prevention and screening services integrated within HIV care at Kenya’s largest national referral hospital found relatively low uptake among WLHIV and identified key barriers to utilisation. In our study population, less than half of WLHIV had screened for cervical cancer despite implementation of integrated cervical cancer screening since 2009. This is higher than previous studies in Nairobi (19.0%)^18^ and a recent review of screening coverage rates in SSA which found ranges from 2.0% to 20.0% in urban areas and 0.4% – 14.0% in rural areas,^19^ but lower than South Africa where cervical cancer screening rates were 54.0%.^19^ The higher screening rate in South Africa could be the result of the implementation of approaches where WLHIV self-collected their samples for examination.^20^ Our results add to growing evidence that more efforts are needed to improve integrated delivery of cervical cancer screening within HIV care and to increase utilisation of services among WLHIV in SSA, a population disproportionately affected by cervical cancer. Further, periodic audits of screening activities and monitoring of facilities for quality assurance could help improve screening practices. In Kenya, the cervical cancer screening is higher (14%) among WLHIV compared to the HIV-negative women (3%), based on prior studies.^14,21^ This may be attributed to higher engagement in care, with the majority of WLHIV on ART which requires routine visits to health facilities for medication refills.

In our study, the obstacles to screening utilisation included fear of painful pelvic procedures associated with VIA and Pap smears, avoidance of invasive procedures, encountering male healthcare providers when prompted to screen, and interference with future fertility, similar to other studies.^22,23^ Utilising novel approaches to cervical cancer screening with self-sampling may increase acceptability of cervical cancer screening in this setting. The WHO recommends molecular testing for HPV as primary screening for cervical cancer in low- and middle-income countries using platforms such as Xpert HPV^®^ which has better sensitivity than cytology and VIA, even with self-sampling.^24,25^ To date, Xpert HPV^®^ has not been evaluated as a strategy to improve cervical cancer screening utilisation in Kenya and could be one approach to addressing implementation barriers identified in our study. However, the governments’s support is required in the implementation and sustainability of this novel approach. This will ensure access to free or subsidised HPV screening among WLHIV. The fear of screening, fatalism and fertility concerns can be addressed through routine health education, peer to peer education, counselling to address psychological barriers and media campaigns similar to what has been used to encourage populations to screen for HIV.^26^ General anxiety tools such as a questionnaire need to be considered as they can play vital roles in assessment of fear, anxiety and concerns, thus ensuring these barriers are addressed promptly.^22^ Having an STI in the last 6 months was not significantly associated with cervical cancer screening contrary to other studies that have been conducted in the region. However, diagnostic testing for STIs was not conducted in our studies and STIs rates are likely higher.

Similar to prior studies,^27^ we found that older age and higher education were associated with increased likelihood of screening. Older women with HIV have accessed the health system longer than younger women and may be more sensitised to cervical cancer, thus increasing their likelihood of screening compared to younger women.^28^ Our findings support that sensitisation of cervical cancer prevention and screening implementation should be prioritised for AGYW living with HIV with interventions to raise awareness tailored to this population. There was no difference in screening frequency among married and unmarried WLHIV which differs from prior studies among African women.^29^ In Kenya, women from some regions marry at earlier ages and therefore married women may reflect a younger age group than prior studies outside of Kenya.^30^ Cost was also a barrier to utilising cervical cancer services, especially HPV vaccination. There is a need to address modifiable factors contributing to low uptake of prevention services among young women and those with financial constraints. Cost of the HPV vaccination was reported as the key barrier to accessing the vaccine and no eligible study participants had been vaccinated. These findings are comparable with those of women recruited in a community-based cervical cancer screening programme in rural western Kenya where screening and HPV vaccination uptake was poor.^31^ One approach to reducing barriers to HPV vaccination uptake among young Kenyan WLHIV is offering free or incentivised cytology (when indicated) and vaccination options combined with targeted health education.^32^ The KEN-SHE study evaluated single-dose HPV vaccine efficacy among AGYW in Africa and has the potential to guide public health policy and increase HPV vaccine coverage by reducing the number of required doses.^33^

Provision of free services does not guarantee increased uptake, particularly in SSA. Other additional costs such as transport to the health facility, lost wages and childcare cost could explain why free services are still resulting in poor uptake.^28^ Women with lower social economic status have also been shown to have a negative attitude towards cervical screening even if freely offered, thus warranting the need for more health information to the specific target groups based on factors like particular age or social economic status before implementation of the interventional strategies.^34^ Introducing HPV vaccination programmes for all children aged 9–15 years, ideally before sexual debut, would be especially useful for preventing HPV.

Our study has limitations. Our funding allowed us to hire only one RA to recruit participants which limited our ability to enrol all eligible clients and extended our recruitment period to attain the minimum required sample size. However, consecutive sampling was used and this limitation likely did not bias our results.

We did not distinguish between screening via VIA or Pap smear when ascertaining screening utilisation, so we were unable to determine the factors associated with each approach. Although representative of WLHIV receiving HIV care at the CCC, our sample included only a limited number of adolescents and therefore our results specific to that age group (e.g. HPV vaccine uptake) should be interpreted with caution. We did not ascertain timing or frequency of prior cervical cancer screening. These data would help elucidate the proportion of WLHIV who meet the national guidelines for cervical cancer screening. We only asked if participants were screened for cervical cancer at the CCC and not within a certain timeframe. It is possible that screening could have taken place at another facility outside of the study site. However, our eligibility criteria for participants ≥ 25 years included being a patient at the hospital for ≥ 1 year. The satisfaction with the HCW’s counselling was based on the current HIV clinic visit; however, we did systematically ascertain reasons why participants were not satisfied with the counselling they received. Anecdotally, participants reported that incomplete information was provided on the availability of prevention services. This study was conducted at a single referral hospital in an urban setting and hence may not be generalisable to Kenyan WLHIV in rural settings. Additionally, self-reported data may be influenced by social desirability and recall bias. Our study did not interview the healthcare providers or other stakeholders in the programme who play a key role in the service provision and sustainability.

## Conclusion

In this mixed methods evaluation of cervical cancer screening utilisation among WLHIV receiving HIV care at Kenya’s national referral hospital, utilisation was relatively low. Barriers to utilisation such as fear of painful invasive procedures could potentially be addressed by integrating self-sampling approaches to cervical cancer screening such as Xpert HPV^®^. Negative attitudes towards screening could be addressed by awareness campaigns via mass media. There is a need to improve screening recommendations offered by HCW to WLHIV at diagnosis, ideally at each healthcare visit to avoid missed opportunities.

## APPENDIX 1: Questions used to assess knowledge, attitudes and practices about

1. Have you ever heard of cervical cancer? □ Yes □ No
2. How did you know about cervical cancer? □ Healthcare provider □ Media □ Friends □ School
  If yes to number 1 above, what causes cervical cancer? Have you ever screened for cervical cancer in KNH CCC? □ Yes □ No □ Not sure
3. If you have ever screened for cervical cancer at KNH, what method was used? □ Via villi □ Pap smear □ HPV molecular □ Do not know
4. Were you able to access your cervical cancer screening results? □ Yes □ No
5. If yes to 5 above, how long did it take to get your screening results? □ 0–2 weeks □ 1 month □ 2 months □ 3–6 months □ Did not get results
6. If you have ever screened for cervical cancer, did you know after how long you should screen again? □ Yes □ No
7. Do you know how to prevent yourself from cervical cancer? □ Yes □ No
8. Have you ever heard of HPV vaccine? □ Yes □ No
9. Have you ever received a HPV vaccine? □ Yes □ No
10. If yes to 10 above, how many jabs of the HPV vaccine did you receive? ……………………………
11. Who should provide the HPV vaccine? □ Government □ Self payment □ Paid for by the society □ National hospital insurance □ Do not know
12. Have you ever been told by your healthcare provider, about cervical cancer preventive services when attending your routine care at KNH CCC? □ Yes □ No
13. If yes to 13 above, how would you rate the quality of the information provided by the healthcare providers on cervical cancer preventive services? (1) Poor (2) Average (3) Good (4) Excellent
14. Were you referred to a different unit, away from KNH CCC for the cervical cancer screening or cancer preventive services? □ Yes □ No
15. If you accessed cervical cancer screening or preventive services at KNH CCC, what were the factors, facilitators or motivators that made it easy for you to access these services?
16. Are there challenges that you encountered as you tried to access the cervical cancer preventive services?
17. If you have never been able to access the cervical cancer preventive services, what are the main reasons as to this?
